# Safety and Feasibility of Microporous Polysaccharide Hemosphere Absorbable Hemostat in Robotic Liver Resection: A Single-Center Retrospective Cohort Study

**DOI:** 10.7759/cureus.97384

**Published:** 2025-11-20

**Authors:** Takahisa Fujikawa, Yusuke Uemoto, Kei Harada, Keiji Nagata

**Affiliations:** 1 Surgery, Kokura Memorial Hospital, Kitakyushu, JPN

**Keywords:** absorbable hemostat, bleeding complication, fibrin sealant patch, microporous polysaccharide hemosphere, robotic liver resection

## Abstract

Background: Numerous absorbable hemostats are often employed in liver surgery; however, the safety and efficacy of microporous polysaccharide hemosphere (MPH) absorbable hemostats in robotic liver resection (RLR) remain uncertain.

Methods: This study included 141 cases that underwent RLR at Kokura Memorial Hospital between September 2021 and May 2025. The included patients were divided into three groups: those without using any hemostat (non-hemostat (non-HS) group, n=47), those with the use of fibrin sealant patch (FSP) hemostat (TachoSil®; CSL Behring K.K., Tokyo, Japan) (FSP group, n=23), and those with the use of MPH hemostat (Arista™ AH Absorbable Hemostat; Becton, Dickinson and Company, Franklin Lakes, USA) (MPH group, n=71). The background characteristics and perioperative outcomes were compared between the groups.

Results: There were no differences in any background characteristics. The rate of anatomical hepatectomy in the FSP group was higher than in the MPH or non-HS groups (95.7% vs. 35.2% vs. 36.2%, p<0.001), and surgical blood loss was less in the MPH and non-HS groups than in the FSP group (11 mL vs. 0 mL vs. 34 mL, p<0.001). There were no conversions to open hepatectomy in the MPH group, no serious postoperative complications such as hemorrhage or bile leakage, and no mortality. There were no differences in the time for MPH application between the anterolateral and posterosuperior segment areas (35 sec vs. 36 sec, p=0.396), but a significant difference was observed between FSP and MPH application time in anatomical hepatectomy (382 sec vs. 36 sec, p<0.001).

Conclusions: Application of MPH is easy and effective for hemostasis without increasing the rate of bleeding complications after RLR. In addition, MPH may not raise the risk of bile leakage or intra-abdominal abscess. Thus, the use of MPH for RLR appears to be safe, effective, and time-efficient.

## Introduction

Postoperative bleeding is a significant cause of severe morbidity following liver resection, and perioperative bleeding and blood transfusion significantly increase the rates of mortality and major morbidity [1]. Effective management of bleeding has been shown to lower the risk of complications and subsequent mortality [2]. Therefore, minimizing bleeding during liver resection and managing it adequately are critical. Even with precise surgical methods and sophisticated instruments, oozing of the blood can occur from the cut surface of the liver, particularly when resection results in a deep fissure. Several topical hemostatic agents, including oxidized regenerated cellulose powder, fibrin sealant solution or patch, and microporous polysaccharide hemosphere (MPH) powder, are commonly used with different biologic, chemical, and physical mechanisms of action [3]. Due to their ease of application, these agents have been adopted for widespread use in both open and minimally invasive abdominal surgeries [4,5]. The use of topical hemostatic agents can reduce blood loss and the need for blood transfusion, both of which are associated with adverse events and substantial cost [6,7].

Arista™ AH Absorbable Hemostat (Becton, Dickinson and Company, Franklin Lakes, USA) is an MPH powder made from a plant source that is biologically inert, absorbs fully in 24 to 48 hours, and has no animal or human elements. It has been used in various surgical fields, including general surgery, and has been shown to reduce intraoperative and postoperative bleeding [8-10]. Furthermore, it does not appear to represent a nidus for infection or granuloma formation, and no adverse events have been reported in clinical studies [9,11].

Robotic surgery has become widespread in various fields, and robotic liver resection (RLR) is also being performed in several selected institutions; RLR offers reduced blood loss, a shorter recovery period, and a lower incidence of postoperative complications in comparison to traditional open surgery [12,13]. While some research has examined the efficiency and safety of topical hemostatic agents in open general surgery [10,14-16], there is a notable lack of studies in the context of robotic abdominal surgery, including RLR.

This study aimed to evaluate the safety and feasibility of MPH use in RLR. The authors declare no conflicts of interest.

## Materials and methods

For this retrospective cohort study, we obtained approval from the Institutional Review Board of Kokura Memorial Hospital (approval no. 25022751), and the single institution's prospectively gathered surgery database was checked for potentially pertinent instances. We included a total of 141 consecutive RLRs, performed from September 2021 to May 2025, after excluding cases of emergency surgery and those with synchronous surgery for other malignancies in the current study. The exclusion criteria for RLR included patients with liver tumors measuring 10 cm or larger, as well as those with tumors deemed suitable for vascular reconstruction or multi-visceral resection. All other hepatectomy procedures were planned to be conducted as RLR.

We had generally used a fibrin sealant patch (FSP) (TachoSil®; CSL Behring K.K., Tokyo, Japan) for the purpose of preventing bleeding complications, especially during anatomical hepatectomy, although we currently prefer an MPH application during both anatomical and non-anatomical hepatectomy because of its easy-to-use and secure properties. To analyze the background and surgical factors of the whole cohort, the patients were divided into three groups according to the status of hemostat used: patients with the use of MPH (Arista™ AH Absorbable Hemostat) (MPH group, n=71), those with the use of FSP (TachoSil®) (FSP group, n=23), and those without the use of hemostat (non-hemostat (non-HS) group, n=47). The IWATE criteria were employed on a scale from 0 to 12 to evaluate the difficulty level of RLR [17]. The Clavien-Dindo classification (CDC) was utilized to categorize and evaluate postoperative complications, with complications classified as CDC class 3a or higher deemed significantly important [18]. Operative mortality refers to the incidence of death occurring within 30 days following a surgical procedure.

Statistical analysis

Continuous values were expressed as mean (standard deviation) or median (range), while categorical variables were presented as absolute numbers and percentages. For univariate comparisons, Fisher's exact probability test was used to evaluate categorical variables; alternatively, continuous variables were analyzed by one-way analysis of variance and Kruskal-Wallis tests for normally and non-normally distributed data, respectively. All p-values were two-sided, and p-values less than 0.05 were considered statistically significant. All statistical analyses were performed with EZR (Saitama Medical Center, Jichi Medical University, Saitama, Japan), which is a graphic user interface for R version 2.13.0 (R Foundation for Statistical Computing, Vienna, Austria) [19].

Surgical technique

All RLRs in the present cohort were conducted utilizing the Da Vinci Xi surgical system (Intuitive Surgical, Inc., Sunnyvale, USA). The positioning of the patient and the placement of ports during RLR have been previously detailed [20,21]. Prior to parenchymal transection, the Pringle maneuver was systematically prepared using an extraperitoneal tourniquet system incorporating a Nelaton catheter (Izumo Health Co., Nagano, Japan), as outlined in a previous study [22], and applied as needed. Monopolar curved scissors (Intuitive Surgical, Inc., Sunnyvale, USA) or the double bipolar method were typically employed for adhesiolysis or dissection around the liver. In contrast, the saline-linked coagulation (SLiC) method, which utilized monopolar curved scissors or Maryland bipolar forceps (Intuitive Surgical, Inc., Sunnyvale, USA) in conjunction with saline, was exclusively applied during liver parenchymal transection [20,21].

**Figure 1:**
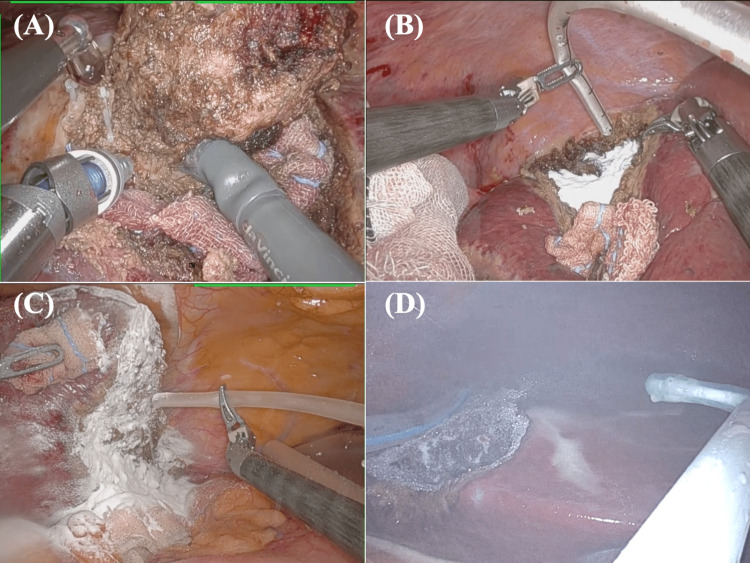
The process of robotic liver resection with the application of MPH (Arista™ AH Absorbable Hemostat). (A) Both anatomical and non-anatomical RLR could be safely performed using the SLiC method, not only in the anterolateral segment area but also in the complicating posterosuperior segment area. (B, C) In the final step of RLR, MPH was applied on the liver parenchymal resection surface (B: status post non-anatomical S7 hepatectomy; C: status post anatomical S2 subsectionectomy). (D) After the MPH application, a coat of anti-adhesive was applied adjacent to the resection surface. MPH, microporous polysaccharide hemosphere; RLR, robotic liver resection; SLiC, saline-linked coagulation

Typical RLR cases with the use of MPH are shown in Figure 1 and Video 1. Both anatomical and non-anatomical RLR could be safely performed using the SLiC method, not only in the anterolateral segment (ALS) area but also in the complicating posterosuperior segment (PSS) area (Figure 1A). Following liver parenchymal resection, hemostasis was achieved using either bipolar or monopolar techniques, followed by a comprehensive visual examination to ensure the absence of any controllable bleeding. After confirming the absence of active bleeding, topical hemostatic agents were applied to the resected liver surface. In the MPH group, MPH was applied on the liver parenchymal resection surface via a sterile malleable 14 Fr. suction catheter (Suction Catheter Suffeed, 500 mm in length; Terumo, Tokyo, Japan) attached to the applicator (Figures 1B-1C). After the MPH application, a coat of anti-adhesive was applied around the liver and hepatoduodenal ligament, including the area adjacent to the resection surface (Figure 1D).

**Video 1:** The process of robotic liver resection with the application of MPH (Arista™ AH Absorbable Hemostat). MPH, microporous polysaccharide hemosphere

## Results

Table 1 displays the patient and tumor characteristics in the current cohort. There were no differences in any background characteristics, including the status of liver function and the type of disease. In the FSP group, the tumor was larger than in the MPH or non-HS groups (32 mm vs. 25 mm vs. 25 mm, p=0.017), although the number of tumors was similar between the groups.

**Table 1:** Patient, laboratory, and tumor characteristics in the current cohort. Bold values indicate statistical significance. HS, hemostat; FSP, fibrin sealant patch; MPH, microporous polysaccharide hemosphere; BMI, body mass index; ICG-R15, indocyanine green retention test after 15 min; HCC, hepatocellular carcinoma; CRCLM, colorectal cancer liver metastasis

Variables	Non-HS (n=47)	FSP (n=23)	MPH (n=71)	P-value
Age, years, median (range)	73 (44-85)	76 (60-86)	76 (29-89)	0.401
Male gender, n (%)	35 (74.5)	19 (82.6)	50 (70.4)	0.509
BMI, kg/m^2^, median (range)	23.9 (16.2-39.8)	23.2 (17.4-34.4)	23.6 (15.7-34.5)	0.736
Platelet count, x10^4^/μL, median (range)	15.9 (6.9-36.7)	15.9 (9.1-30.9)	15.3 (7.0-33.1)	0.618
ICG-R15, %, median (range)	11.2 (1.9-43.2)	14.1 (4.4-36.0)	13.5 (1.5-63.2)	0.489
Disease: HCC, n (%)	25 (53.2)	11 (47.8)	39 (54.9)	0.977
Disease: CRCLM, n (%)	16 (34.0)	9 (39.1)	24 (33.8)	0.977
Number of the tumor, median (range)	1 (1-4)	1 (1-3)	1 (1-3)	0.888
Maximum size of the tumor, mm, median (range)	25 (10-80)	32 (13-60)	25 (10-120)	0.017

Table 2 displays the surgical factors and patient outcomes in the current cohort. Although the rates of repeat hepatectomy or resection of the PSS area were similar between the groups, the rate of anatomical hepatectomy (95.7% vs. 35.2% vs. 36.2%, p<0.001) and the difficulty score (8 vs. 5 vs. 5, p<0.001) in the FSP group were significantly higher than in the MPH or non-HS groups. Accordingly, the operative time (492 min vs. 295 min vs. 259 min, p<0.001) and console time (403 min vs. 217 min vs. 155 min, p<0.001) were longer in the FSP group than in the other groups. Surgical blood loss was less in the MPH and non-HS groups than in the FSP group (11 mL vs. 0 mL vs. 34 mL, p<0.001). There was no conversion to open hepatectomy in the current cohort.

**Table 2:** Surgical factors and outcomes in the current cohort. Bold values indicate statistical significance. HS, hemostat; FSP, fibrin sealant patch; MPH, microporous polysaccharide hemosphere; PSS, posterosuperior segments; PHLF, post-hepatectomy liver failure; CDC, Clavien-Dindo classification; LOS, length of postoperative stay; NA, not applicable

Variables	Non-HS (n=47)	FSP (n=23)	MPH (n=71)	P-value
Repeat hepatectomy, n (%)	11 (23.4)	5 (21.7)	24 (33.8)	0.35
Anatomical hepatectomy, n (%)	17 (36.2)	22 (95.7)	25 (35.2)	<0.001
Resection of PSS area, n (%)	20 (42.6)	12 (52.2)	33 (46.5)	0.747
Difficulty score, median (range)	5 (1-11)	8 (5-11)	5 (1-10)	<0.001
Operative time, min, median (range)	259 (90-690)	492 (302-716)	295 (95-574)	<0.001
Console time, min, median (range)	155 (30-616)	403 (245-653)	217 (35-497)	<0.001
Blood loss, mL, median (range)	0 (0-400)	34 (0-204)	11 (0-180)	<0.001
Red blood cell transfusion, n (%)	0 (0.0)	0 (0.0)	1 (1.4)	0.609
Conversion to open surgery, n (%)	0 (0.0)	0 (0.0)	0 (0.0)	NA
PHLF (Grade B, C), n (%)	0 (0.0)	0 (0.0)	0 (0.0)	NA
Operative mortality, n (%)	0 (0.0)	0 (0.0)	0 (0.0)	NA
Postoperative complication (CDC 3a or higher), n (%)	1 (2.1)	2 (8.7)	0 (0.0)	0.043
LOS, days, median (range)	8 (4-22)	9 (7-48)	8 (5-28)	0.006

Concerning the postoperative complications, there were three severe complications (CDC 3a or higher) that occurred in the current cohort (one postoperative abdominal bleeding requiring re-laparotomy in the non-HS group, one bile leakage requiring percutaneous drainage, and one small bowel obstruction requiring surgical adhesiolysis in the FSP group). Otherwise, there were no cases of grade B or C liver failure and no mortality in the whole cohort. The length of postoperative hospital stay was shorter in the MPH and non-HS groups than in the FSP groups (eight days vs. eight days vs. nine days, p=0.006). In the MPH group, neither postoperative intra-abdominal bleeding nor bile leakage/intra-abdominal abscess occurred in the current cohort.

**Figure 2:**
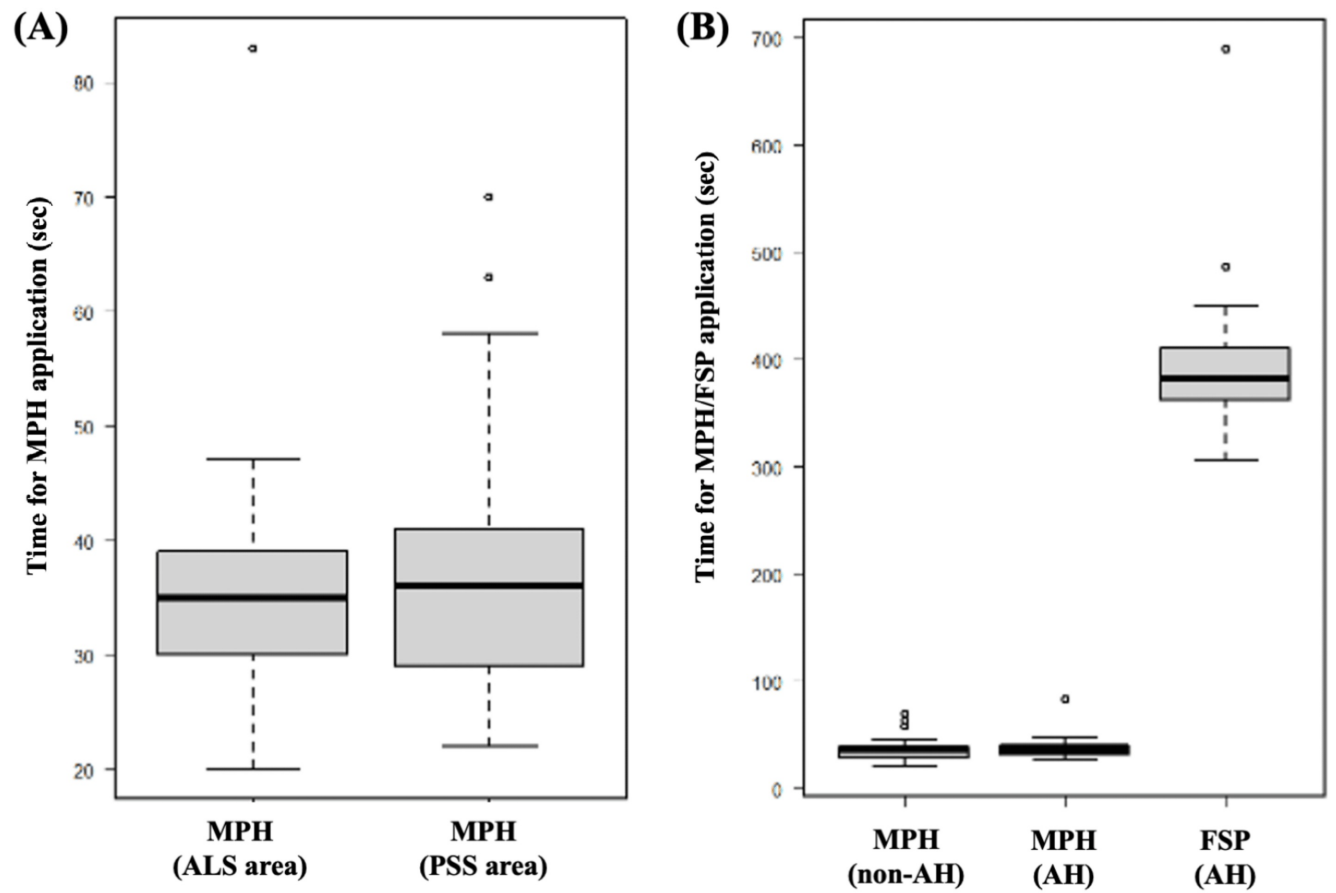
Comparison of time for hemostat application with reference to the type of hemostat and area of application. (A) There was no difference in the time for MPH application between the ALS area and PSS area (35 sec vs. 36 sec, p=0.396). (B) There was no difference in the MPH application time between non-anatomical and anatomical hepatectomy (36 sec vs. 36 sec, p=0.423), although a significant difference was observed between FSP and MPH application time in anatomical hepatectomy (382 sec vs. 36 sec, p<0.001). MPH, microporous polysaccharide hemosphere; ALS, anterolateral segments; PSS, posterosuperior segments; AH, anatomical hepatectomy; FSP, fibrin sealant patch

Table 3 and Figure 2 show the comparison of time for hemostat application with special reference to the type of hemostat and area of application. Although the PSS area was deeper and was under limited access, there were no differences in the time for MPH application between the ALS and PSS areas (35 sec vs. 36 sec, p=0.396) (Figure 2A). Concerning the anatomical hepatectomy, there was also no difference in the MPH application time between non-anatomical and anatomical hepatectomy (36 sec vs. 36 sec, p=0.423), although a significant difference was observed between FSP and MPH application time in anatomical hepatectomy (382 sec vs. 36 sec, p<0.001) (Figure 2B).

**Table 3:** Comparison of time for hemostat application with reference to the type of hemostat and area of application. * indicates statistical significance. MPH, microporous polysaccharide hemosphere; PSS, posterosuperior segments; ALS, anterolateral segments; FSP, fibrin sealant patch

Variables	Time for application, sec, median (range)
MPH application in the PSS area (n=33)	36 (22-70)
MPH application in the ALS area (n=38)	35 (20-83)
MPH application after non-anatomical hepatectomy (n=46)	36 (20-70)
MPH application after anatomical hepatectomy (n=25)	36* (26-83)
FSP application after anatomical hepatectomy (n=23)	382* (305-690)

## Discussion

In the current study, we evaluated the safety and efficacy of using MPH in both non-anatomical and anatomical RLR. In the case of the MPH application, there was no conversion to open hepatectomy, no severe postoperative complications (such as postoperative bleeding or bile leakage), no cases of post-hepatectomy liver failure, and no mortality. Regarding the operability and time efficiency, there were no differences in the time for MPH application between the ALS and PSS areas, but a significant difference was observed in the time between MPH and FSP application during anatomical hepatectomy. Thus, the application of MPH is convenient and effective for hemostasis without increasing the rate of postoperative bleeding complications or intra-abdominal abscesses. The use of MPH in RLR is considered safe, effective, and time-efficient.

MPH enhances endogenous coagulation activities by absorbing water and low molecular weight substances from the blood to concentrate platelets and clotting proteins at its beaded surface. It is considered physiologically safe, fully absorbed within 24 to 48 hours, and devoid of human or animal elements. MPH has been used in various fields of surgery and has been shown to reduce intraoperative and postoperative bleeding [8-10]. Regarding liver resection, one report suggested the effectiveness of MPH in open liver surgery [10], although there is scarce evidence concerning its safety and efficacy in robotic or laparoscopic hepatectomy. In the current study, no significant increase was observed in the incidence of bile leakage, intra-abdominal abscess, or postoperative bleeding events.

We had generally used FSP for the purpose of preventing bleeding complications during anatomical hepatectomy. However, due to its equivalent efficacy and safety as well as easier-to-use property, we currently prefer MPH application during both anatomical and non-anatomical hepatectomy. FSP is a strong hemostatic agent utilized in liver parenchymal transection [23,24]. In the context of open liver surgery, the application of FSP is comparatively straightforward; however, it becomes technically difficult and time-consuming during laparoscopic or robotic hepatectomy due to the delicate and sticky nature of FSP and the motion restrictions of the instruments. The current study suggested that in the case of MPH application during RLR, there were no severe postoperative complications, such as postoperative bleeding or bile leakage, and no mortality. There were no differences in the time for MPH application between the ALS and PSS areas, but a significant difference was observed between the time for FSP and MPH application during anatomical hepatectomy. Accordingly, the MPH application appears to be as safe and effective as FSP, less time-consuming, and easier to handle than FSP.

Regarding the intraperitoneal adhesions after abdominal surgery, a number of topical hemostatic agents have been studied for their anti-adhesive properties [25], although some of these agents may themselves promote adhesion formation. Hoffmann et al. evaluated the impact of several commonly used hemostatic agents on adhesion formation in a rat peritoneal model, demonstrating that MPH was the most effective at limiting adhesion formation compared to other hemostatic agents, and it showed no residual agent or no difference in acute inflammation when compared to controls [25]. Our preliminary clinical data also indicated favorable outcomes regarding its anti-adhesive effect in RLR (data not shown). It is suggested that MPH could be used not only to promote hemostasis but also to reduce adhesions during minimally invasive hepatectomy. Additional laboratory and human trials are needed for a definite conclusion.

Limitations of the study

There are certain limitations of the current investigation. First, the study's retrospective design limits its relevance in determining how treatments affect outcomes. Second, the sample size was modest; a bigger sample size would likely produce more trustworthy recommendations. Third, there was a group imbalance in the current research; the FSP group included more complex cases, which may have affected operative outcomes. Nonetheless, we are convinced that this strategy is a step in the right direction toward RLR's eventual standardization. It could lead to improved security and outcomes and be a further step toward the safe and efficient mainstream use of MPH during RLR.

## Conclusions

Application of MPH during RLR appears to be safe and feasible even in the deep PSS area. RLR combined with the MPH application can be effective for hemostasis without increasing the rate of postoperative bleeding complications or intra-abdominal abscess. The use of MPH in RLR appears to be safe, effective, and time-efficient.
